# Extracellular NLRP3 inflammasome particles are internalized by human coronary artery smooth muscle cells and induce pro-atherogenic effects

**DOI:** 10.1038/s41598-021-94314-1

**Published:** 2021-07-26

**Authors:** Susanne Gaul, Karen Marie Schaeffer, Lena Opitz, Christina Maeder, Alexander Kogel, Luisa Uhlmann, Hermann Kalwa, Ulf Wagner, Jan Haas, Amirhossein Behzadi, Pablo Pelegrin, Jes-Niels Boeckel, Ulrich Laufs

**Affiliations:** 1 Klinik und Poliklinik für Kardiologie, Universitätsklinikum Leipzig, Leipzig University, Johannisallee 30, 04103 Leipzig, Germany; 2 Medical Faculty, Rudolf-Boehm-Institut für Pharmakologie und Toxikologie, Leipzig University, Leipzig, Germany; 3 Klinik für Gastroenterologie, Hepatologie, Infektionsmedizin, Rheumatologie, Universitätsklinikum Leipzig, Leipzig, Germany; 4 Department of Internal Medicine III, University of Heidelberg, Heidelberg, Germany; 5 DZHK (German Centre for Cardiovascular Research), Heidelberg/Mannheim, Germany; 6 Biomedical Research Institute of Murcia (IMIB-Arrixaca), Clinical University Hospital Virgen de La Arrixaca, Murcia, Spain

**Keywords:** Pattern recognition receptors, Immunology, Cell death and immune response, Cell biology, Cell adhesion, Cell migration, Cell signalling, Mechanisms of disease

## Abstract

Inflammation driven by intracellular activation of the NLRP3 inflammasome is involved in the pathogenesis of a variety of diseases including vascular pathologies. Inflammasome specks are released into the extracellular compartment from disrupting pyroptotic cells. The potential uptake and function of extracellular NLRP3 inflammasomes in human coronary artery smooth muscle cells (HCASMC) are unknown. Fluorescently labeled NLRP3 inflammasome particles were isolated from a mutant NLRP3-YFP cell line and used to treat primary HCASMC for 4 and 24 h. Fluorescent and expressional analyses showed that extracellular NLRP3-YFP particles are internalized into HCASMC, where they remain active and stimulate intracellular caspase-1 (1.9-fold) and IL-1β (1.5-fold) activation without inducing pyroptotic cell death. Transcriptomic analysis revealed increased expression level of pro-inflammatory adhesion molecules (*ICAM1*, *CADM1*), *NLRP3* and genes involved in cytoskleleton organization. The NLRP3-YFP particle-induced gene expression was not dependent on NLRP3 and caspase-1 activation. Instead, the effects were partly abrogated by blocking NFκB activation. Genes, upregulated by extracellular NLRP3 were validated in human carotid artery atheromatous plaques. Extracellular NLRP3-YFP inflammasome particles promoted the secretion of pro-atherogenic and inflammatory cytokines such as CCL2/MCP1, CXCL1 and IL-17E, and increased HCASMC migration (1.8-fold) and extracellular matrix production, such as fibronectin (5.8-fold) which was dependent on NFκB and NLRP3 activation. Extracellular NLRP3 inflammasome particles are internalized into human coronary artery smooth muscle cells where they induce pro-inflammatory and pro-atherogenic effects representing a novel mechanism of cell-cell communication and perpetuation of inflammation in atherosclerosis. Therefore, extracellular NLRP3 inflammasomes may be useful to improve the diagnosis of inflammatory diseases and the development of novel anti-inflammatory therapeutic strategies.

## Introduction

Sterile inflammation drives the pathogenesis of various diseases and is controlled by intracellular multiprotein inflammasome complexes, such as the nod-like receptor family pyrin domain-containing 3 (NLRP3) inflammasome that can be activated by danger-associated molecular patterns (DAMPs) and pathogen-associated molecular patterns (PAMPs)^1,2^. Activated NLRP3 aggregates with the adaptor protein apoptosis-associated speck-like protein (ASC) and pro-caspase-1 forming an NLRP3 inflammasome speck, that cleaves pro-interleukin-1β into its mature form^3^. Caspase-1 also cleaves and activates the pore protein Gasdermin D that targets the plasma membrane forming pores that allow the release of mature IL-1β as well as induce a specific type of cell death called pyroptosis^4^. Pyroptotic cell death has been described in myeloid cells such as monocytes and macrophages but also occurs in vascular endothelial cells where it triggers the adhesion of monocytes^5^. NLRP3 inflammasome-mediated pyroptosis has been shown to be elevated in atherosclerotic plaques^6^ where it correlates with plaque instability and vascular inflammation, indicating that pyroptosis plays an important role in the progression of atherosclerosis. Interestingly, pyroptosis also occurs in ischemic disease settings such as myocardial infarction (MI)^7–9^. Inhibition of NLRP3 activation or pyroptosis by pharmacological or genetic interventions appears cardioprotective in *in-vitro* and *in-vivo* studies^6,8,10–14^. Of note, besides cytokines, pyroptotic cells release functional NLRP3 inflammasome specks into the extracellular compartment where they remain active ^15,16^. Previous studies by our group and others have reported a crucial role of NLRP3 inflammasome activation and its release during the development and progression of chronic diseases in non-cardiovascular settings such as non-alcoholic steatohepatitis (NASH)^17^ and kidney injury^2^. We demonstrated that non-myeloid cells such as hepatocytes undergo pyroptotic cell death and release NLRP3 inflammasomes into extracellular space where they can be phagocytosed by neighboring hepatic stellate cells leading to their cell activation and promoting liver fibrogenesis^17^. As macrophages in the vessel wall and smooth muscle cells are in close proximity, extracellular NLRP3 inflammasome particles released by pyroptotic macrophages^18^ may affect smooth muscle cell function. The physiologic and potentially pathophysiologic roles of these extracellular NLRP3 inflammasome particles in primary human coronary artery smooth muscle cells have not been studied and therefore represent a novel research field of systemic cell-cell communication during inflammation.

Therefore, we hypothesized that human smooth muscle cells internalize extracellular NLRP3 inflammasome particles which may lead to increased pro-inflammatory signaling, cell migration and extracellular matrix production.

## Methods

The data that support the findings of this study are available from the corresponding author upon reasonable request. Please see the resources table in the Supplementary Information.

### Cell culture

Primary human coronary artery smooth muscle cells (HCASMC) were purchased from Lonza and cultured in growth media containing SmBM Basal Medium (CC-3181, Lonza) and SmGM-2 SingleQuots supplements (CC-4149) required for growth of smooth muscle cells. THP1 monocytic cell line (DSMZ, Cat.No: ACC16) was cultured in RPMI 1640 medium supplemented with 10% (v/v) FCS, 100 U/ml penicillin and 100 mg/ml streptomycin.

### NLRP3 mutant cell line and NLRP3- YFP inflammasome particle isolation

A stable mutant NLRP3 (p.D303N)-YFP HEK cell line was used to isolate constitutively active YFP-labelled NLRP3 inflammasome oligomeric particles as previously described^15,17,19^. As a control, WT HEK 293 cells were applied to the same particle isolation protocol. Isolated mutant NLRP3-YFP inflammasome oligomeric particles were measured on a fluorometer and counted in a hemocytometer using a fluorescence microscope and used to stimulate HCASMC for 4 h and 24 h.

### Immunofluorescent analysis

Internalization of extracellular NLRP3-YFP inflammasome particles in target cells was determined by immunofluorescent staining of YFP-tagged NLRP3 particles and cell organelle staining. HCASMC were seeded on a black 96-well plate with clear flat bottom (Corning) (5 × 10^3^ cells/ well). After treatment with extracellular NLRP3-YFP inflammasome particles (3 NLRP3-YFP particles per seeded cell; 3:1) for 4 h, cells were washed three times with PBS and fixed in 4% paraformaldehyde (PFA) for 15 min at room temperature (RT) followed by permeabilization with PBS + 0.25% Trion-X100 for 10 min at RT in the dark. After washing, cells were blocked with 1% bovine serum albumin (BSA) in PBS for 30 min at RT protected from light. Anti-YFP antibody (1:200, 2.5 µg/ml, Abcam) or IgG rabbit control (2.5 µg/ml, Dianova) were diluted in DAKO Antibody diluent (DAKO) and incubated over night at 4 °C. Fluorescently-labeled anti-rabbit Alexa 647 secondary antibody (1:1000, ThermoFisher Scientific) was diluted in DAKO antibody diluent and incubated for 1 h at RT in the dark. After washing, cytoskeleton staining was performed by incubating the cells with 200 nM working solution of Actin-stain Phalloidin 555 for 30–60 min at RT in the dark. Nuclei were stained with DAPI or Hoechst 33,342 (1:200, 0.5% v/v) in PBS. Finally, cells were washed and covered with PBS for visualization on a ZEISS Axiovert 200 M fluorescent microscope. Z-stacking (12 subsequently optical slices of 0.8 µm thickness) were performed with AxioVision LE Rel 4.4 software. A minimum of five random selected fields were used. An ImageStreamX Mk II Imaging Flow Cytometer was used as another method for internalization studies.

### Confocal microscopy of internalized NLRP3-YFP particles

HCASMC were seeded at a density of 1 × 10^4^ cells per cm^2^ on 25-mm coverslips 24 h before the start of the experiment. HCASMC were treated with extracellular NLRP3-YFP inflammasome particles for 4 h (3:1 particles/ cell). Coverslips were then washed with HBS and incubated with Cell Mask Deep Red plasma membrane stain (Invitrogen, Carlsbad, California, United States) for 10 min at 37 °C according to the manufacturer's protocol. Coverslips were mounted and imaged using a Leica DMI8/SP8 confocal laser scanning microscope (Leica Microsystems, Wetzlar, Germany). Pinhole adjustment was set to 1 Airy unit. For YFP excitation a 488 nm and for CellMask Deep Red plasma membrane stain a 638 nm Laser was used. Emission filters were adjusted accordingly. Images were analyzed using LasX (Leica Microsystems, Wetzlar, Germany) and ImageJ.

### Cell stimulation

All cells were kept at 5% CO_2_ and 37 °C. HCASMCs were treated with isolated NLRP3-YFP inflammasome particles (3:1 NLRP3-YFP particles/ cell) for 4 h (internalization, gene expression, migration, western blot) and 24 h (gene expression, extracellular matrix production). For mechanistic studies, NLRP3 inhibitor MCC950 (1 µM, Invivogen), caspase-1 inhibitor Ac-YVAD-cmk (25 µg/ml, Invivogen) and NFκB inhibitor IKK-16 (2 µM, Selleckchem) were added to the media 30 min before treatment. Endocytosis inhibitor Cytochalasin D (4 µM, Invitrogen) was added 30 min before treatment with NLRP3-YFP particles and was washed off 3 times by washing with culture medium.

### LDH cell death assay

Supernatant of extracellular NLRP3-YFP inflammasome particle-treated HCASMC was collected and used for cytotoxicity analysis using the Pierce LDH Cytotoxicity Assay (Pierce, ThermoFisher Scientific) according to the manufacturer´s instructions. OD value of LDH positive control was set 100% and % of LDH release in sample supernatant was calculated accordingly.

### Gene expression

RNA was isolated using RNeasy Mini kit from Qiagen and reverse transcription was performed with iScript (Biorad) Mastermix. Primer sequences and probes are listed in the Major Resources Table in the Supplemental Material. qPCR analyses were performed with PowerUp SYBR Green Mastermix (ThermoFisher) or TaqMan Fast Advanced Master-Mix (Applied Biosystems). All samples were run in duplicates and relative gene expression was converted using the 2-ΔΔCT method against the mean of two internal control housekeeping genes *RPLP0* and *Tata-box binding protein* (TBP) or *β-2 Mikroglobulin* (*B2M*) for human. ΔΔCT = (CTexperiment gene – CT mean experiment housekeeping)—(CTcontrol gene – CT mean control housekeeping). The relative gene expression in the control group was set at 1.

### RNA sequencing

Briefly, total RNA was isolated from HCASMC (N = 2) using the Qiagen miRNeasy Mini Kit. All samples had > 100 ng of input RNA and were of excellent quality (RIN = 10). Sequencing libraries were created using the TruSeq Stranded Total RNA kit, utilizing random hexamer primer to enable the analysis of coding and non-coding RNA species after removal of cytoplasmic rRNA. RNA sequencing was performed on a NovaSeq 6000 using 2 × 150 bp end technology. The Bioconductor packages edgeR and limma were used to normalize sequence count data and conduct differential gene expression analysis^20,21^. Genes that were below detection limit were set 0.1. Differentially expressed genes (DEGs) specific for NLRP3-YFP treated HCASMC cells were subjected to validation using RT-PCR (N = 3–6). Gene Set Enrichment Analysis^22^ were conducted with the 200 most up-regulated genes (mean log2 fold > 3.5) using EnrichR^23^ and NCATS BioPlanet 2019 comprehensive integrated pathway analysis. We used NCBI’s Gene Expression Omnibus (GEO) database^24^ for searching of relevant studies and then retrieved a specific dataset from Ayari et al.^25^ where 34 patients underwent carotid endarterectomy to collect atheroma plaque (ATH) and the macroscopically intact tissue. Transcriptional profiling was based on Affymetrix Human GeneChip Gene 1.0 ST microarray (Affymetrix, Santa Clara, CA, USA)^25^. The re-analysis of this pre-existing microarray dataset limited our assay costs, the supply of biological material and the efficacy of a replication-based strategy. DEGs found by RNA Seq of HCASMC treated with extracellular NLRP3 inflammasome particles were validated and the gene expression compared between atheroma plaque and adjacent macroscopically intact tissue from the same patient (N = 34).

### Immunoblot analysis

HCASMC were homogenized in RIPA buffer (Cell Signaling, USA) containing protease inhibitor cocktail HALT (ThermoFisher, USA). For immunoblot analysis 20–30 µg of protein lysate was resolved on Any kD Mini-PROTEAN TGX Precast polyacrylamide gels (Biorad, Hercules, CA, USA), transferred to nitrocellulose membrane, blocked in 5% Blotting-grade blocker (Biorad) and incubated with appropriate primary antibodies. Anti-IL1β (1:1000, Abcam), anti-caspase-1 p20 Bally-1 (Adipogen), anti-NLRP3 NBP1 (1:1000, NBP1-77,080 Novus Biological), anti-ASC AL177 (1:1000, Adipogen), anti-Gasdermin D (L60) (1:1000, Cell Signaling) and anti-β-Actin (1:10,000, Abgent) were incubated over-night. β-Actin was used for normalization. Membranes were incubated with peroxidase-conjugated secondary antibody (DAKO, USA). Protein bands were visualized with the enhanced chemiluminescence (Pico or Femto, Pierce ThermoFisher Scientific, Waltham, MA USA) reagent and digitized using iBright FL1500 Imaging System (ThermoFisher, USA). Densitometric analysis was performed on background subtracted blots using Image J.

### Cell migration

Migration was analyzed in Boyden chambers (HCASMC: 8 µm pore size) using QCM Chemotaxis Cell Migration Assay (Merck). Inserts were coated with sterile-filtered 10 µg/ml Typ 1 collagen (Corning Collagen Typ I rat) and 0.2 × 10^6^ cells were seeded on the insert in serum-starved media with 0.2% FCS (SFM). The well was filled with serum-containing media (10% FCS). PDGF (10 ng/ml, Peprotech) was used as positive control, Actinomycin D (5 µg/ml, Merck) as negative control. Extracellular NLRP3-YFP inflammasome particles were added to the cells (3:1 particles/ cell) in the insert for 4 h at 37 °C. Then, migrated cells of the lower side of the membrane were stained with cell stain crystal violet and remaining cells in the insert were removed with a Q-tip. After washing, stained cells on the apical side of the porous membrane were lysed with extraction buffer and the absorbance was measured at 560 nm in a 96 well plate.

### Scratch assay

HCASMC (1.0 × 10^4^ cells/well) were seeded in 8 replicates onto 96-well IncuCyte ImageLock tissue culture plates (Essen BioScience, #4379) and incubated for 72 h to form a cell monolayer, before being treated with NLRP3-YFP inflammasome particles (3:1 particles/ cell) for 4 h. PDGF (10 ng/ml) and Actinomycin (5 µg/ml) were incubated for 24 h as positive and negative controls. Wounds were made with the 96-well WoundMaker (Essen BioScience, #4493). The plate was washed twice to remove any detached cells. Images of the wounds were automatically acquired within the CO_2_ incubator using the IncuCyte ZOOM software package (Essen BioScience, #2016A). Typical kinetic updates were taken at 0 and after 24 h. Wound confluency [%] was calculated using the IncuCyte ZOOM software. Cell count that migrated into the scratch were counted manually.

### Extracellular matrix (ECM) production

To analyze cell-free ECM production in HCASMC, an established protocol was used^26^. Therefore, cells (5 × 10^4^ cells) were seeded on glass coverslips and cultured overnight until treated with extracellular NLRP3-YFP inflammasome particles (3:1 particles/ cell) for 24 h. As positive control, TGFβ1 (10 ng/ml) was stimulated for 48 h. To exclude potential contamination of ECM extracts with cell surface and intracellular proteins, cells were removed by incubating the wells with ammonium hydroxide (20 mM) for 5 min followed by washing with de-ionized water. Afterwards, ECM was fixed with 2% PFA for 10 min at RT and blocked with 1% BSA/PBS. Antibody against fibronectin (1 µg/ml, Santa Cruz) or IgG mouse isotype control was solved in DAKO antibody diluent (DAKO) and incubated overnight at 4 °C in the dark. Anti-mouse Cy3 antibody (1:1000, Dianova) was diluted in DAKO antibody diluent and incubated for 1 h at RT. To check for successful cell removal, wells were incubated with Hoechst 33,342 (0.5% v/v) in PBS and incubated for 5 min at 37 °C. Cells treated without ammonium hydroxide were run in parallel as positive control. ECM was analyzed on a Keyence BZ-X810 all-in-one fluorescence microscope. Four random selected fields were used for ImageJ analysis. Percentage of positive stained area from images with same magnification was calculated.

### IL-1β ELISA

Supernatant of HCASMC were used for the analysis of released IL-1β using the Human IL-1 beta/IL-1F2 Quantikine ELISA Kit (R&D, DLB50).

### Human proteome profiler cytokine array

The Proteome Profiler Human Cytokine Array Kit (R&D) is a membrane-based sandwich immunoassay and was used for the detection of 36 cytokines, chemokines, and acute-phase proteins, simultaneously. Therefore, supernatant of NLRP3-YFP particle-treated HCASMC (4 h) and untreated control in culture medium (N = 3) was used according to manufacturers instructions. Captured proteins were visualized using chemiluminescent detection reagents with short and long-time exposure. Profiles of mean spot pixel density was created using ImageJ analysis software 1.53e after background subtraction. Only membranes with the same exposure were used for comparisons.

### Statistics

Statistical analyses were performed with Graph Pad Prism (version 7; Graph Pad Software Inc., La Jolla, CA, USA). The significance level was set at p < 0.05 for all comparisons. Gaussian distributed data were analyzed using one-way analysis of variance and Sidak post-hoc test or uncorrected Fishers LSD test. Nonparametric gene expression data were log2-transformed and compared by ANOVA and appropriate post-hoc test. Two groups were analyzed by Student´s T-test. Data are expressed as mean ± standard error of mean (SEM).

## Results

*Extracellular NLRP3 inflammasome particles are internalized by human coronary artery smooth muscle cells (HCASMC) and lead to increased caspase-1 and IL-1β activation without inducing pyroptotic dell death.*

**Figure 1:**
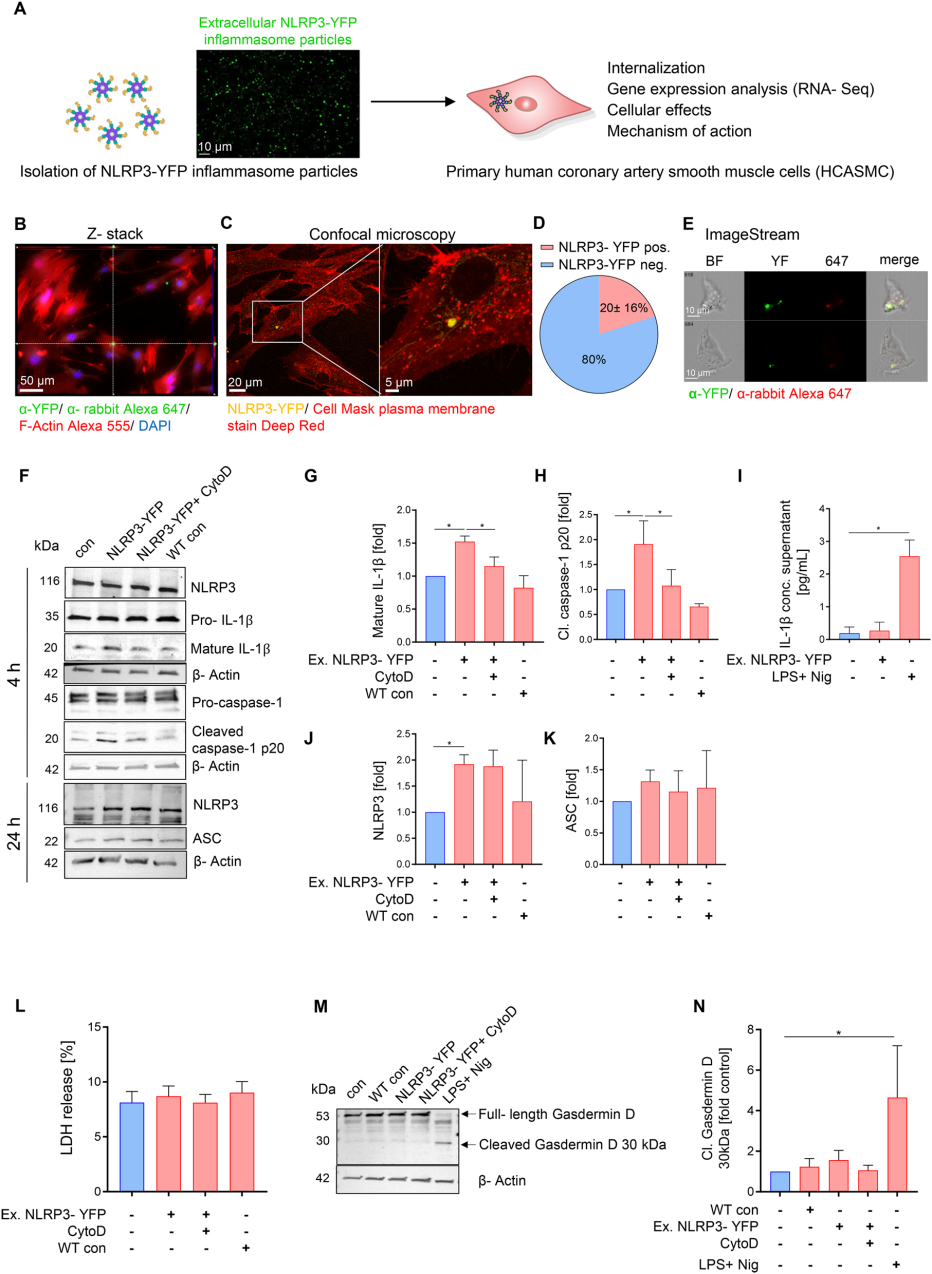
Extracellular NLRP3-YFP inflammasome particles are internalized by primary human coronary artery smooth muscle cells (HCASMC) and induce caspase-1 and IL-1β activation. **(A)** Schematic overview of study objective. Constitutively active mutant HEK NLRP3-YFP (p.D303N) cells were used for isolation of cell-free NLRP3-YFP inflammasome particles that are used for treatment of HCASMC (scale bar: 10 µm). (**B)** Internalization of extracellular NLRP3-YFP inflammasomes in HCASMC after 4 h of incubation determined by immunofluorescent staining (N = 3) with an anti-YFP antibody or IgG isotype control and fluorescently-labeled anti-rabbit Alexa 647 secondary antibody (scale bar: 50 µm). Alexa Fluor 555 Phalloidin and DAPI were used for F-Actin and nucleus staining, respectively. Z-stacks with xz and yz focal planes (white dashed lines) showing internalized NLRP3-YFP inflammasome in HCASMC. (**C)** Internalization of extracellular NLRP3-YFP inflammasomes (purified particles) (yellow). HCASMC were incubated for 4 h. Uptake was confirmed via confocal microscopy. Plasma membrane staining (red) was carried out using Cell Mask Deep Red (scale bar: 20 µm). Micrographs depicting internalization and subcellular localisation of NLRP3-YFP inflammasome particles are shown in overview and enhanced magnification (scale bar: 5 µm). (**D)** For quantification, four representative fields of view were analyzed. (**E)** Representative ImageStream analysis of HCASMC showing internalized extracellular NLRP3-YFP inflammasome (scale bar: 10 µm) by merging the brightfield image (BF), YFP signal of the NLRP3-YFP inflammasome and the immunostained signal of the anti-YFP/ anti-rabbit Alexa 647 antibody. **(F-K)** HCASMC were treated with extracellular NLRP3-YFP inflammasome particles (3:1) for 4 h and 24 h with our without pre-incubation of Cytochalasin D (CytoD, 4 µM) for 30 min and total protein lysate was used for immunoblotting. Immunoblot (N = 4) of (**F)** NLRP3, pro-IL-1β and mature IL-1β 17 kDa, pro-caspase-1 and activated caspase-1 p20 (4 h) as well as endogenous NLRP3 and ASC after 24 h. The corresponding densitometric analysis of (**G)** mature cleaved IL-1β and (**H)** activated caspase-1 p20 as well as **(I)** IL-1β secretion (pg/ml) is shown (after 4 h). As positive control for endogenous inflammasome activation and IL-1β secretion, cells were stimulated with LPS (1 µg/ml, 3 h) and Nigericin (10 µM, 90 min). (**J, K)** Densitometric analysis of NLRP3 and ASC protein level after 24 h of stimulation with extracellular NLRP3-YFP inflammasome particles (N = 4). β-Actin was used as housekeeping control. To exclude effects of particle isolation, same protocol for inflammasome particle isolation was performed on WT HEK cells and were used for treatment (WT con) of HCASMC. Data were normalized on β-Actin and set at 1. (**L)** Membrane disruption was measured by the release of lactate dehydrogenase (LDH) into supernatant of HCASMC treated with NLRP3-YFP inflammasome particles (3:1 particles/cell) for 4 h. LDH values were normalized to positive LDH control which was set 100%. Immunoblot (N = 3) of (**M)** Gasdermin D (full-length 53 kDa) and its active cleaved N-terminal fragment (30 kDa). β-Actin was used as housekeeping control. (**N)** Densitometric analysis of the cleaved Gasdermin D fragment (30 kDa) was performed and normalized on untreated control which was set at 1. HCASMC treated with LPS + Nig were used as positive control. Differences between the groups were analysed by One-way ANOVA and uncorrected Fishers LSD post-hoc test (*p < 0.05).

To determine whether extracellular NLRP3 inflammasome particles can be internalized by HCASMC, constitutively active, recombinant fluorescently labeled NLRP3 inflammasome particles were isolated from mutant NLRP3-YFP (p.D303N) cells (Fig. 1A)^15,19^. After fixation and immunostaining of HCASMC, NLRP3-YFP inflammasome internalization was confirmed after 4 h of incubation. F-Actin and nucleus staining was used to identify intracellular focal planes in a Z-stack analysis. NLRP3-YFP signal in the same focal planes with F-Actin and DAPI were considered intracellular (Fig. 1B). As control, NLRP3-YFP inflammasome treated cells were incubated with IgG isotype control and fluorescent secondary antibody. To validate internalization of NLRP3-YFP inflammasome particles in HCASCM, confocal microscopy on live cells stimulated for 4 h with extracellular NLRP3-YFP particles was performed. Plasma membrane was stained with Cell Mask Deep Red to prove intracellular localisation (Fig. 1C). NLRP3-YFP positive cells were counted and normalized to total cell count per field (N = 4). We found that 20 ± 16% of HCASMC ingested extracellular NLRP3-YFP inflammasome particles after 4 h (Fig. 1D). Using the ImageStream instrument as another method to prove internalization, we also detected NLRP3-YFP signals in HCASMC after 4 h of incubation (Fig. 1E).

To determine whether internalized NLRP3-YFP inflammasome particles remain active in HCASMC, intracellular activation of caspase-1 (p20), as well as IL-1β production and activation (cleaved 17 kDa form) were quantitated in lysates of HCASMC after 4 h of treatment with extracellular NLRP3-YFP inflammasome particles (Fig. 1F–H). Active IL-1β was increased 1.5-fold and caspase-1 1.9-fold compared to control while the pro-form of caspase-1 and IL-1β was not altered (Fig. 1G, H). Interestingly, the secretion of mature IL-β was not altered after stimulation with extracellular NLRP3-YFP particles while the classcial inflammasome activation with LPS + Nig increased IL-1β release from HCASMC even though to a small extent (Fig. 1I). To test whether activation of caspase-1 and IL-1β was dependent on internalized extracellular NLRP3-YFP inflammasome particles, HCASMC were pre-treated with the endocytosis inhibitor Cytochalasin D for 30 min. Cytochalasin D reduced the production of cleaved caspase-1 and IL-1β in HCASMC lysates (Fig. 1F–H). To exclude particle isolation related effects, we also tested particles isolated from HEK WT cells (WT con) which showed no effect on caspase-1 and IL-1β activation (Fig. 1F–H). Endogenous level of NLRP3 protein was increased (1.9-fold) after 24 h in HCASMC after treament with NLRP3-YFP particles while no changes were observed after 4 h (Fig. 1F, J). ASC protein was only mildly increased but did not reach significance (Fig. 1F, K). Further, internalized extracellular NLRP3-YFP particles did not induce cell death or cytotoxic effects (Fig. 1L) in HCASMC nor activated Gasdermin D in HCASMC after 4 h of treatment (Fig. 1M, N**)** indicating that internalized NLRP3 inflammasome particles did not induce pyroptotic cell death. Similar effects were found when HCASMC were treated with ASC-GFP specks from activated THP1 monocytes (Supplementary Figure S1). ASC-GFP specks were internalized in HCASMC measured by confocal imaging and induced IL-1β cleavage intracellularly (Supplementary Figure S1). To test whether the effect on IL-1β release of extracellular NLRP3-YFP particles is different between HCASMC and macrophages, which are classical phagocytic cells, human THP1-differentiated macrophages were stimulated with extracellular NLRP3-YFP inflammasome particles and IL-1β secretion were found to be increased in resting macrophages which was further amplified after LPS priming (Supplementary Figure S2). This suggests that extracellular NLRP3 inflammasome particles play an important role in cell-cell communication and perpetuation and amplification of inflammation.

### Extracellular NLRP3 inflammasome particles regulate gene expression in HCASMC that are associated with plaque formation

**Figure 2:**
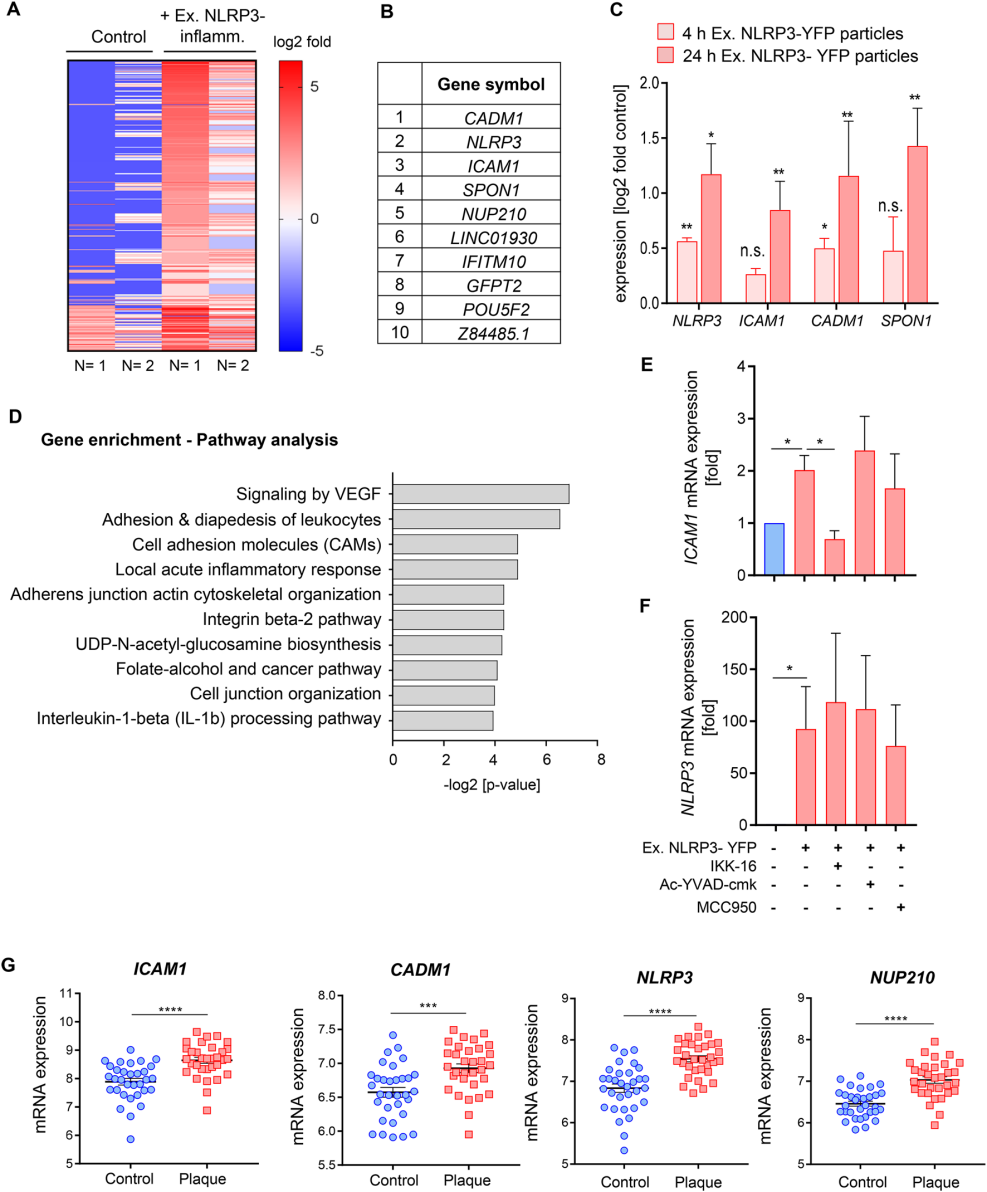
Extracellular NLRP3-YFP inflammasome particles regulate gene expression in HCASMC. **(A)** Heatmap of genes upregulated in HCASMC treated with extracellular NLRP3-YFP inflammasome particles (N = 2) for 24 h. (**B)** List of the ten most upregulated genes. (**C)** Validation of four listed genes (*NLRP3*, *ICAM1*, *CADM1*, *SPON1*) using qPCR after 4 and 24 h treatment with extracellular NLRP3-YFP particles. Data were normalized on the mean of two housekeeping genes (*RPLP0* and *TBP*) and were log2-transformed for equal distribution. Untreated cells (control) were set at 1. (**D)** Gene enrichment analysis of the 200 most upregulated genes (mean log2 fold > 3.5 vs. control) was performed using EnrichR and NCATS BioPlanet 2019 comprehensive integrated pathway analysis. Pathways with *p* < 0.05 are shown. mRNA expression of (**E)** adhesion molecule *ICAM1* and (**F)** *NLRP3* in HCASMC (N = 9) treated with extracellular NLRP3-YFP inflammasomes (3:1 particles/ cell) for 24 h with or without pre-incubation of caspase-1 inhibitor (Ac-YVAD-cmk, 25 µg/ml), NLRP3 inhibitor (MCC950, 1 µM) and NFκB inhibitor (IKK-16, 2 µM). Data are expressed as mean ± SEM and were normalized on the mean of two housekeeping genes (*RPLP0* and *TBP*) and referenced to untreated control which was set at 1**.** Differences between the groups were calculated using One-way ANOVA and uncorrected Fisher´s LSD post-hoc test (*p < 0.05). (**G)** Selected genes (*NLRP3*, *ICAM1*, *CADM1*, *NUP210*) were re-analysed using a pre-existing microarray dataset and the gene expression was compared between atheroma plaque and adjacent macroscopically intact tissue from the same patient (N = 34). Data are expressed as mean ± SEM and differences between the groups were calculated using unpaired, two-tailed Student´s T-Test (***p < 0.001, ****p < 0.0001).

The gene expression in HCASMC after treatment with extracellular NLRP3 inflammasome particles was determined using RNA transcriptomic analysis. 1247 genes were differentially expressed in extracellular NLRP3 inflammasome treated cells compared to untreated control (> 2.5-fold induction). Regulated genes were selected by expression level and regulation (Fig. 2A). The ten most upregulated genes are listed in Fig. 2B. A subset of four genes were selected for validation in qPCR analysis including *ICAM1* (**p < 0.01), *CADM1* (*p < 0.05), *NLRP3* (*p < 0.05) and *SPON1* (**p < 0.01) (Fig. 2C) and were analyzed after 4 and 24 h. To gain a better sense of biological functions represented by the differentially expressed genes, we performed a gene enrichment analysis of the 200 most upregulated genes and selected the pathways with an enrichment combined score 30 and a p-value ≤ 0.05 (Fig. 2D). The analysis identified ten annotations with enrichment scores > 30. Most of the up-regulated pathways were associated with leukocyte adhesion and VEGF signaling as well as cytoskeleton organization and signaling involved in acute inflammatory response and IL-1β processing (Fig. 2D). To investigate whether the upregulation of selected genes after internalization of extracellular NLRP3 inflammasome particles in HCASMC was dependent on increased NLRP3 and caspase-1 activation or induction of NFκB activation, HCASMC were co-stimulated with the caspase-1 inhibitor Ac-YVAD-cmk, the NLRP3 inhibitor MCC950 or the NFκB inhibitor IKK-16. While the upregulation of the adhesion molecule *ICAM1* in HCASMC treated with extracellular NLRP3-YFP inflammasome particles were attenuated when cells were co-stimulated with the NFκB inhibitor IKK-16, NLRP3 gene expression was not affected by NFκB inhibition. Further, inhibition of NLRP3 and caspase-1 activity did not prevent from upregulation of *ICAM1* and *NLRP3* gene expression after 4 (data not shown) and 24 h (Fig. 2E, F). This indicates that the effect of extracellular NLRP3 inflammasome particles on *NLRP3* and *ICAM1* in HCASMC is not dependent on NLRP3 and caspase-1 activation but partly through activation of NFκB signaling.

Datasets from patients with atherosclerotic plaques and healthy vessels (N = 34)^25^ showed increased expression levels of *ICAM1*, *CADM1*, *NLRP3* and *NUP210 in* human atherosclerotic plaques compared to intact controls (Fig. 2G). This suggests that extracellular inflammasomes in atherosclerotic plaques may induce gene expression changes that are relevant for plaque formation and progression.

### Extracellular NLRP3 inflammasome particles stimulate the secretion of pro-atherogenic cytokines and promote smooth muscle cell migration which is dependent on NFκB and NLRP3 inflammasome activation

**Figure 3:**
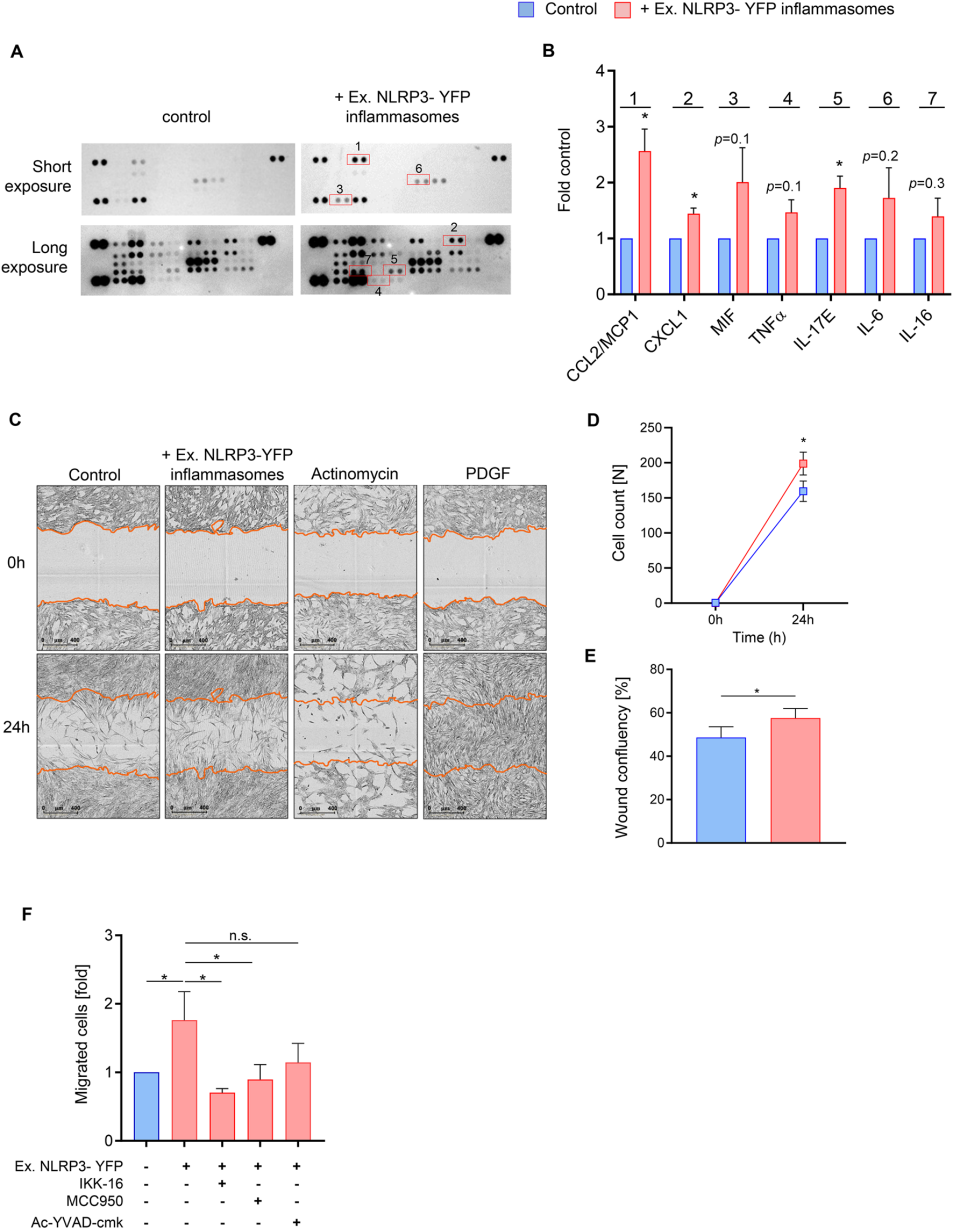
Extracellular NLRP3-YFP inflammasome particles promote release of pro-atherogenic cytokines and cell migration in HCASMC. **(A, B)** Supernatant of HCASMC (N = 3) after 4 h treatment with extracellular NLRP3-YFP inflammasomes (3:1 particles/cell) were used for the human proteome profiler cytokine array. Modified cytokines/chemokines were surrounded with a red square and labelled (1–7). Membranes were applied for short and long-time exposure. (**B)** Densitometric analysis of the labelled cytokines (1–7) after background subtraction was performed. Data were normalized on supernatant from control cells which was set at 1. (**C)** Scratch assay of HCASMC treated with NLRP3-YFP inflammasome particles (3:1 particles/ cell) for 4 h. PDGF (10 ng/ml) and Actinomycin (5 µg/ml) were stimulated for 24 h. Representative images of wounds at the beginning (0 h) and after 24 h are shown. Wounds were automatically acquired within the CO_2_ incubator using the IncuCyte ZOOM software package. (**D)** Cell count [N] at 0 h and after 24 h within the scratch and (**E)** wound confluency [%] was calculated using the IncuCyte ZOOM software. Data are expressed as mean ± SEM and differences between the groups were calculated using unpaired, two-tailed Student´s T-Test (*p < 0.05). (**F)** Smooth muscle cell migration was studied in a Boyden chamber by adding NLRP3-YFP inflammasome particles (3:1 particles/ cell) to HCASMC for 4 h and analyzing the migrated cells on the lower side of the insert membrane which was stained with crystal violet and measured at 560 nm in a fluorometer (N = 4–6). Differences between the groups were analysed by One-way ANOVA and uncorrected Fishers LSD post-hoc test (*p < 0.05).

To determine whether cytokines or chemokines, other than IL-1β, might be upregulated after treatment with extracellular NLRP3-YFP inflammasome particles, supernatant of NLRP3-YFP treated cells were applied to the human proteome cytokine profiler array kit which enables the detection of 36 cytokines, chemokines and acute-phase proteins, simultaneously. Interestingly, we identified several cytokines and chemokines to be upregulated, such as CCL2/MCP1 (2.6-fold, p < 0.05), CXCL1 (1.5-fold, p < 0.05), MIF (2.0-fold, p = 0.1), TNFα (1.5-fold, p = 0.1), IL-17E (1.9-fold, p < 0.05), IL-6 (1.7-fold, p = 0.2) and IL-16 (1.4-fold, p = 0.3) (Fig. 3A, B). All of them are associated with pro-inflammatory and pro-atherogenic signaling pathways. As some of them are also potent drivers of cell migration, we investigted whether extracellular NLRP3-YFP inflammasome particles promote cell migration and asked whether this is dependent on NFκB and NLRP3 inflammasome activation. Cell migration is a crucial step during atherosclerosis initiation and progression^27^. The migratory capacity was either tested in a scratch assay using the Incucyte System or in a Boyden chamber with a membrane of defined pores (8 µm). For the scratch assay, HCASMC were incubated with extracellular NLRP3-YFP inflammasome particles for 4 h and then washed off and were grown for further 24 h. Phase-contrast images were automatically taken by the Incucyte system from every well at 0 and after 24 h (Fig. 3C). Cell count [N] (Fig. 3D) and wound confluency [%] (Fig. 3E) of the scratch after 24 h were significantly increased when HCASMC were pre-treated with extracellular NLRP3-YFP inflammasomes. For the analysis in a Boyden chamber, HCASMC were treated with extracellular NLRP3-YFP inflammasome particles (3:1, particles/cell) and transmigration of cells was analyzed after 4 h (Fig. 3F). HCASMC showed a 1.8-fold increased transmigratory capacity after 4 h of stimulation with extracellular NLRP3-YFP inflammasome particles compared to untreated control cells which were abrogated when cells were co-incubated with inhibitors against NFκB and NLRP3 activation (Fig. 3F). Blocking caspase-1 activity by AC-YVAD-cmk showed a trend toward reduced cell migration but did not reached significance. These data suggest that both, NFkB and NLRP3 activation, affect migratory capacity of smooth muscle cells and is induced by treatment with extracellular NLRP3-YFP inflammasome particles promoting atherogenesis and plaque formation.

### Treatment of HCASMC with extracellular NLRP3 inflammasome particles promote cell-free extracellular matrix (ECM) production

**Figure 4:**
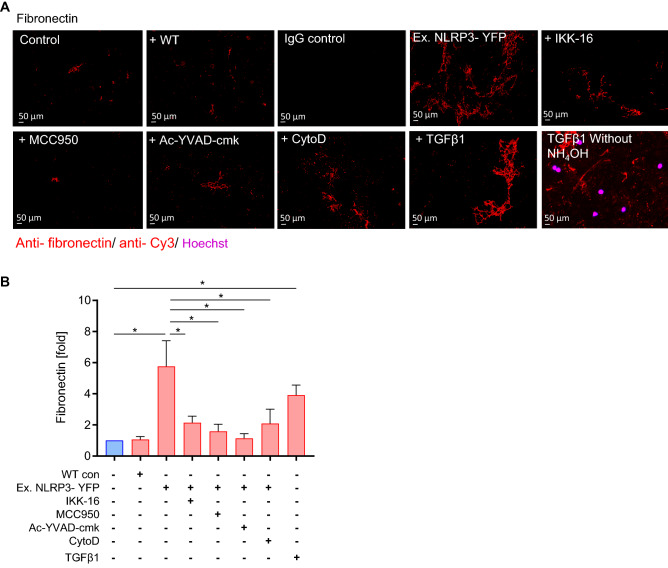
Extracellular NLRP3-YFP inflammasome particles induce extracellular matrix (ECM) production in HCASMC that can be reduced by different inhibitors. **(A)** Immunofluorescent staining of cell-free ECM protein fibronectin (red) produced by HCASMC after treatment with extracellular NLRP3-YFP inflammasomes (3:1 particles/ cell) for 24 h (N = 5). ECM production was reduced when cells were co-stimulated with inhibitors such as IKK-16 (2 µM), Ac-YVAD-cmk (25 µg/ml), MCC950 (1 µM) for 24 h or pre-incubation with CytoD (2.5 µg/ml) for 30 min. Antibody against fibronectin (1 µg/ml, Santa Cruz) or IgG mouse isotype control as well as anti-rabbit anti-mouse Cy3 antibody (1:1000, Dianova) were used (scale bar: 50 µm). Ammonium hydroxide (NH_4_OH) was used to remove cells and only stain extracellular fibronectin. Negative Hoechst (nucleus) staining showed successful removal of cells. TGFβ1 (48 h) was used as positive control. (**B**) Five random selected fields were used for ImageJ analysis and the positive stained area (% of area) from images with same magnification was calculated. Data were normalized on the untreated control which was set at 1. Data are expressed as mean ± SEM and differences between the groups were compared using One-way ANOVA and Sidak´s post-hoc test (*p < 0.05).

Smooth muscle cells produce ECM that positively correlates with lesion progression^27^. Whereas most of the ECM within a healthy artery contains type I and type III fibrillar collagen, atherosclerotic lesions exhibit high levels of proteoglycans composed of type I collagen fibrils and fibronectin^28^. To study whether extracellular NLRP3 inflammasomes affect ECM production, HCASMC were treated with extracellular NLRP3-YFP inflammasome particles for 24 h (3:1 particles/ cell) and cell-free derived ECM fibronectin was determined as described previously^26^. As positive control, cells were stimulated with TGFβ1 for 48 h. After treatment, HCASMC were incubated with ammonium hydroxide to remove cells and exclude potential contamination of ECM extracts with cell surface and intracellular proteins. Cell removal was confirmed by negative Hoechst (nucleus) staining (Fig. 4A). Treatment of HCASMC with extracellular NLRP3-YFP inflammasome particles promoted production of ECM fibronectin by 5.8-fold (Fig. 4B). When co-stimulated with inhibitors against NFκB, NLRP3 and caspase-1 activation the production of extracellular fibronectin was abrogated (Fig. 4A, B). Inhibition of endocytosis via cytochalasin D also reduced the NLRP3-YFP mediated fibronectin production. However, we cannot exclude whether cytochalasin D itself affected ECM production as it affects actin microfilaments.

## Discussion

Emerging evidence indicates that inflammasome specks are released from pyroptotic cells^15,16,29^ and that pyroptotic cell death is increased in *in-vivo* models of cardiovascular diseases^11,18,30^. While the intracellular NLRP3 inflammasome activation is well characterized, the extracellular function of NLRP3 inflammasomes is less studied and data on their physiological and pathophysiological function on human vascular cells are missing^16,31^. Previous studies demonstrated an important role of smooth muscle cells in the development and progression of atherosclerotic lesion formation^32^. Our study demonstrates that extracellular NLRP3 inflammasome specks are ingested by human coronary artery smooth muscle cells and induce pro-inflammatory and pro-atherogenic effects partly by activation of NFkB and upregulation of endogenous NLRP3 expression in recipient cells without inducing cell death. The internalization was confirmed by confocal microscopy of the fluorescent NLRP3 inflammasome particles in HCASMC and by ImageStream analysis and by blocking its internalization with cytochalasin D, an endocytosis inhibitor. The uptake of extracellular NLRP3-YFP particles could have been detected in around 20% of HCASMC. The internalized extracellular NLRP3 inflammasome particles promote intracellular caspase-1 and IL-1β activation in primary HCASMC which indicates that internalized inflammasome specks remain enzymatically active and further perpetuate and amplify inflammatory signaling in recipient cells. IL-1β production was abrogated when endocytosis in HCASMC was blocked. Interestingly, IL-1β activation was only increased intracellularly while no IL-1β secretion could have been detected in supernatant. This was associated with a lack of Gasdermin D activation and LDH release, parameters of pyroptotic cell death. One explanation for this could be that the internalized NLRP3-YFP inflammasome particles only prime the cells by upregulation of NFκB and NLRP3 without inducing substantial pyroptotic cell death or the amount of cleaved Gasdermin D, secreted IL-1β and LDH of NLRP3-YFP positive cells (20%) were too little to detect changes in whole cell supernatants and lysates.

Transcriptomic analysis revealed that inflammatory surface adhesion molecules such as *ICAM1* and *CADM1* but also *NLRP3* were upregulated in HCASMC after treatment with NLRP3 inflammasome particles. Gene enrichment analyses showed that most of the upregulated pathways are related to leukocyte adhesion, acute inflammatory response and IL-1β signaling as well as cytoskeleton organization^22^. These are all processes that play important roles during atherogenesis^33^.

Adhesion and chemoattractive molecules are responsible for the interaction of monocytes with cells of the vascular wall and promote their diapedesis into the intima where they differentiate into macrophages. During atherosclerotic plaque formation, SMCs and macrophages are in direct contact through adhesion molecules such as ICAM1, vascular cell adhesion molecule-1 (VCAM1), and fractalkine (CX3CL1)^34–36^. Our findings demonstrate that extracellular NLRP3 inflammasome particles induce expression of adhesion molecules in smooth muscle cells and also promote the secretion of (C‐C motif) ligand 2 (CCL2) and (C‐X‐C motif) ligand 1 (CXCL1), known to be potent mediators of inflammation, migration and the cellular crosstalk between SMC and monocytes and macrophages^37^. During vascular injury, SMCs in the intima media undergo transformation from a quiescent/contractile phenotype to an active/synthetic phenotype characterized by increased cell migration and secretion of a variety of chemokines such as chemokine CCL2 and CXCL1 which leads to recruitment of monocytes^38,39^. To further study whether the increased mRNA expression of *NLRP3* and adhesion molecule ICAM1 after treatment with extracellular NLRP3 inflammasome particles were dependent on intracellular NLRP3 and caspase-1 activation or NFκB signaling, HCASMC were co-stimulated with the caspase-1 inhibitor Ac-YVAD-cmk, the NLRP3 inhibitor MCC950 and IKK-16. Interestingly, NLRP3 and caspase-1 inhibition did not reduce inflammatory gene expression. Instead, we found that blockade of NFκB signaling by IKK-16 abrogated the effect of extracellular NLRP3 inflammasome particle-induced upregulation of *ICAM1* gene expression but not *NLRP3* speculating that extracellular NLRP3 inflammasome particles might be sensored as danger signals in recipient cells. However, we cannot exclude that also other transcription factors than NFκB are involved in this NLRP3-YFP-mediated regulation of gene expression. This indicates that NLRP3 and caspase-1 activity is not responsible for changes in gene expression after NLRP3-YFP inflammasome treatment. This is in line with previous findings where MCC950 inhibits canonical and non-canonical NLRP3 inflammasome activation but does not reduce gene expression level of inflammasome genes^40^.

Furthermore, our results showed that HCASMC treated with extracellular NLRP3-YFP particles released pro-inflammatory and pro-atherogenic factors other than IL-1β, such as CCL2/MCP1, CXCL1, MIF, IL-17E, TNFα and to a lesser extent IL-6 and IL-16. IL-17E has been described to induce migration and invasion in cancer cells^41^ but also to enhance innate immune response^42^. MIF is secreted in response to diverse inflammatory stimuli, and has been associated with a clear pro-inflammatory and pro-atherogenic role in multiple studies of patients and animal models^43^.

The NLRP3-YFP induced upregulation of these cytokines and chemokines was associated with increased cell migration, a key mechanism during plaque formation. During atherogenesis, SMC migrate from the media into the intima of the vessel wall where they possess unique atherogenic properties^27,32^. Our results showed that extracellular NLRP3 inflammasome particles promoted HCASMC migration which was dependent on NFκB and NLRP3 inflammasome activation. We assume that the interaction of increased NFkB-mediated gene expression and release of pro-migratory and pro-inflammatory cytokines such as CCL2, CXCL1, IL-17E and others are involved in this process.

Smooth muscle cells are the major cell type that produces extracellular matrix, which accumulates over the course of lesion progression. During lesion formation, SMC synthesize higher amounts of proteoglycans such as collagen type 1 and fibronectin, but also proteases and cytokines^44^. Proteoglycans in the vessel wall are able to entrap additional LDL cholesterol leading to further foam cell formation and fueling the vicious cycle^27,32^. Our data demonstrate that extracellular NLRP3 inflammasomes stimulate the production of ECM, such as fibronectin, in HCASMC. This could have been prevented by inhibition of NFκB, NLRP3 and caspase-1 activation suggesting that the production of ECM depends on NFκB-mediated ECM production and the intracellular activation of the NLRP3 inflammasome. However, it is also possible that the effects on ECM production are mediated via the increased secretion of pro-atherogenic cytokines and chemokines induced by NLRP3-YFP inflammasome treatment. Nevertheless, our data support the hypothesis that extracellular NLRP3 inflammasome specks are able to promote smooth muscle cell migration and ECM production contributing to atherogenesis.

In summary, our results show that NLRP3 inflammasome particles, either present in the circulation or in local inflamed tissue, represent a novel danger signal with pro-inflammatory and pro-atherogenic properties that is a novel mechanism of cell-cell communication to maintain the inflammatory vicious cycle. This mechanism may be physiologic during the acute inflammatory response, but under chronic disease conditions, such as in atherosclerosis or after MI, this process fuels inflammation and tissue remodeling. These findings contribute to a better understanding of inflammation in a variety of human diseases by suggesting a novel mechanism of the perpetuation of chronic inflammation. Further elucidation of this concept may lead to improvements in risk stratification and potential anti-inflammatory therapeutic strategies to treat atherothrombosis^45,46^.

## Supplementary Information

Supplementary file.

## Abbreviations

ASC: Apoptosis-associated speck-like protein containing CARD
HCASMC: Human coronary artery smooth muscle cells
HEK: Human embryonic kidney
LDH: Lactate dehydrogenase
LPS: Lipopolysaccharide
Nig: Nigericin
NLRP3: NOD-, LRR-, and pyrin domain-containing protein 3
ICAM1: Intercellular adhesion molecule 1
CADM1: Cell adhesion molecule 1
NUP210: Nucleoporin
SPON1: Spondin1
MI: Myocardial infarction
RT: Room temperature
BSA: Bovine serum albumin
PFA: Paraformaldehyde
PDGF: Platelet-derived growth factor
CCL2/MCP1: CC-chemokine ligand 2
CXCL1: Chemokine (C-X-C motif) ligand 1
MIF: Migration inhibitory factor
TNFα: Tumor necrosis factor α
IL-17E: Interleukin 17 E
CM: Culture medium
SFM: Serum-free medium

## References

[1] Chen GY, Nuñez G (2010). Sterile inflammation: sensing and reacting to damage. Nat. Rev. Immunol..

[2] Zewinger S (2020). Apolipoprotein C3 induces inflammation and organ damage by alternative inflammasome activation. Nat. Immunol..

[3] Schroder K, Tschopp J (2010). The inflammasomes. Cell.

[4] Broz P, Pelegrín P, Shao F (2020). The gasdermins, a protein family executing cell death and inflammation. Nat. Rev. Immunol..

[5] Yin, Y. *et al.* Early hyperlipidemia promotes endothelial activation via a caspase-1-sirtuin 1 pathway. *Arteriosclerosis, Thrombosis Vasc. Biol.* **35,** 804–816 (2015). 10.1161/ATVBAHA.115.305282 PMC4376583 25705917

[6] Duewell P (2010). NLRP3 inflammasomes are required for atherogenesis and activated by cholesterol crystals. Nature.

[7] Lin J (2013). Oxidized low density lipoprotein induced caspase-1 mediated pyroptotic cell death in macrophages: implication in lesion instability?. PLOS ONE.

[8] Kawaguchi M (2011). Inflammasome activation of cardiac fibroblasts is essential for myocardial ischemia/reperfusion injury. Circulation.

[9] Mezzaroma E (2011). The inflammasome promotes adverse cardiac remodeling following acute myocardial infarction in the mouse. Proc. Natl. Acad. Sci..

[10] Gage J, Hasu M, Thabet M, Whitman SC (2012). Caspase-1 deficiency decreases atherosclerosis in apolipoprotein E-null mice. Can. J. Cardiol..

[11] Grebe A, Hoss F, Latz E (2018). NLRP3 inflammasome and the IL-1 pathway in atherosclerosis. Circ. Res..

[12] Liang Y (2019). Colchicine inhibits endothelial inflammation via NLRP3/CRP pathway. Int. J. Cardiol..

[13] Martinez GJ, Celermajer DS, Patel S (2018). The NLRP3 inflammasome and the emerging role of colchicine to inhibit atherosclerosis-associated inflammation. Atherosclerosis.

[14] Merhi-Soussi F (2005). Interleukin-1 plays a major role in vascular inflammation and atherosclerosis in male apolipoprotein E-knockout mice. Cardiovasc. Res..

[15] Baroja-Mazo A (2014). The NLRP3 inflammasome is released as a particulate danger signal that amplifies the inflammatory response. Nat. Immunol..

[16] Franklin BS (2014). The adaptor ASC has extracellular and 'prionoid' activities that propagate inflammation. Nat. Immunol..

[17] Gaul, S. *et al.* Hepatocyte pyroptosis and release of inflammasome particles induce stellate cell activation and liver fibrosis. *J. Hepatol*. **74**, 156–167 (2021). 10.1016/j.jhep.2020.07.041 PMC7749849 32763266

[18] Zhaolin, Z., Guohua, L., Shiyuan, W. & Zuo, W. Role of pyroptosis in cardiovascular disease. *Cell Prolif* **52**, e12563 (2019). 10.1111/cpr.12563 PMC6496801 30525268

[19] Martin-Sanchez, F., Gomez, A.I. & Pelegrin, P. Isolation of Particles of Recombinant ASC and NLRP3. *Bio-protocol* **5**, e1480 (2015). 10.21769/BioProtoc.1480 PMC5659372 29082277

[20] Robinson MD, McCarthy DJ, Smyth GK (2009). edgeR: a Bioconductor package for differential expression analysis of digital gene expression data. Bioinformatics.

[21] Ritchie ME (2015). limma powers differential expression analyses for RNA-sequencing and microarray studies. Nucleic Acids Res..

[22] Subramanian A (2005). Gene set enrichment analysis: a knowledge-based approach for interpreting genome-wide expression profiles. Proc. Natl. Acad. Sci..

[23] Kuleshov MV (2016). Enrichr: a comprehensive gene set enrichment analysis web server 2016 update. Nucleic Acids Res..

[24] Barrett T (2013). NCBI GEO: archive for functional genomics data sets–update. Nucleic Acids Res..

[25] Ayari H, Bricca G (2013). Identification of two genes potentially associated in iron-heme homeostasis in human carotid plaque using microarray analysis. J. Biosci..

[26] Hellewell, A.L., Rosini, S. & Adams, J.C. A Rapid, Scalable Method for the Isolation, Functional Study, and Analysis of Cell-derived Extracellular Matrix. *J Vis Exp ***119**, 55051 (2017). 10.3791/55051 PMC5351878 28117783

[27] Basatemur, G.L., Jorgensen, H.F., Clarke, M.C.H., Bennett, M.R. & Mallat, Z. Vascular smooth muscle cells in atherosclerosis. *Nat. Rev. Cardiol ***12**, 727–744 (2019). 10.1038/s41569-019-0227-9 31243391

[28] Chaher, N., Hajhosseiny, R., Phinikaridou, A. & Botnar, R.M.Imaging the Extracellular Matrix in Prevalent Cardiovascular Diseases. *Appl. Sci. ***10**, 4001 (2020).

[29] Shi J (2015). Cleavage of GSDMD by inflammatory caspases determines pyroptotic cell death. Nature.

[30] Zhang Y (2019). Inflammasome activation promotes deep vein thrombosis through pyroptosis. Blood.

[31] Hoss F, Rodriguez-Alcazar JF, Latz E (2017). Assembly and regulation of ASC specks. Cell Mol Life Sci CMLS.

[32] Doran AC, Meller N, McNamara CA (2008). Role of smooth muscle cells in the initiation and early progression of atherosclerosis. Arterioscler. Thromb. Vasc. Biol..

[33] Ruparelia N, Chai JT, Fisher EA, Choudhury RP (2017). Inflammatory processes in cardiovascular disease. A route to targeted therapies. Nat. Rev. Cardiol..

[34] Braun M, Pietsch P, Schrör K, Baumann G, Felix SB (1999). Cellular adhesion molecules on vascular smooth muscle cells. Cardiovasc. Res..

[35] O'Brien KD (1993). Vascular cell adhesion molecule-1 is expressed in human coronary atherosclerotic plaques. Implications for the mode of progression of advanced coronary atherosclerosis. J. Clin. Investig..

[36] Endres M, Laufs U, Merz H, Kaps M (1997). Focal expression of intercellular adhesion molecule-1 in the human carotid bifurcation. Stroke.

[37] Lim JH, Um HJ, Park J-W, Lee I-K, Kwon TK (2009). Interleukin-1β promotes the expression of monocyte chemoattractant protein-1 in human aorta smooth muscle cells via multiple signaling pathways. Exp. Mol. Med..

[38] Yu, B. *et al.* Vascular Stem/Progenitor Cell Migration Induced by Smooth Muscle Cell-Derived Chemokine (C-C Motif) Ligand 2 and Chemokine (C-X-C motif) Ligand 1 Contributes to Neointima Formation. *Stem Cells* **34**, 2368–2380 (2016). 10.1002/stem.2410 PMC5026058 27300479

[39] Chistiakov DA, Orekhov AN, Bobryshev YV (2015). Vascular smooth muscle cell in atherosclerosis. Acta Physiol. (Oxf.).

[40] Coll RC (2015). A small-molecule inhibitor of the NLRP3 inflammasome for the treatment of inflammatory diseases. Nat. Med..

[41] Guo, N. *et al.* Interleukin-17 Promotes Migration and Invasion of Human Cancer Cells Through Upregulation of MTA1 Expression. *Front Oncol* **9**, 546 (2019). 10.3389/fonc.2019.00546 PMC6596356 31281798

[42] Senra L (2019). IL-17E (IL-25) Enhances Innate Immune Responses during Skin Inflammation. J. Invest. Dermatol..

[43] van der Vorst EPC, Döring Y, Weber C (2015). MIF and CXCL12 in cardiovascular diseases: functional differences and similarities. Front. Immunol..

[44] Gomez D, Owens GK (2012). Smooth muscle cell phenotypic switching in atherosclerosis. Cardiovasc. Res..

[45] Toldo S (2016). Inhibition of the NLRP3 inflammasome limits the inflammatory injury following myocardial ischemia-reperfusion in the mouse. Int. J. Cardiol..

[46] van Hout GPJ (2017). The selective NLRP3-inflammasome inhibitor MCC950 reduces infarct size and preserves cardiac function in a pig model of myocardial infarction. Eur Heart J..

