# Supplementary material for: Extracellular NLRP3 inflammasome particles are internalized by human coronary artery smooth muscle cells and induce pro-atherogenic effects
Source: Sci Rep. 2021 Jul 26;11:15156. doi: 10.1038/s41598-021-94314-1 (PMC8313534; doi:10.1038/s41598-021-94314-1)

Supplementary Information

Extracellular NLRP3 inflammasome particles are internalized by human coronary artery smooth muscle cells and induce pro-atherogenic effects

Susanne Gaul[1*], Karen Marie Schaeffer[1], Lena Opitz[1], Christina Maeder[1], Alexander Kogel[1], Luisa Uhlmann[1], Hermann Kalwa[2], Ulf Wagner[3], Jan Haas[4,5], Amirhossein Behzadi[1], Pablo Pelegrin[6], Jes-Niels Boeckel[1], Ulrich Laufs[1]

[1] Klinik und Poliklinik für Kardiologie, Universitätsklinikum Leipzig, Leipzig University, Leipzig, Germany
[2] Rudolf-Boehm-Institut für Pharmakologie und Toxikologie, Leipzig University, Medical Faculty, Leipzig, Germany
[3] Klinik für Gastroenterologie, Hepatologie, Infektionsmedizin, Rheumatologie, Universitätsklinikum Leipzig, Leipzig, Germany
[4] Department of Internal Medicine III, University of Heidelberg, Heidelberg, Germany
[5] DZHK (German Centre for Cardiovascular Research), Heidelberg/Mannheim, Germany
[6] Biomedical Research Institute of Murcia (IMIB-Arrixaca), Clinical University Hospital Virgen de la Arrixaca, Murcia, Spain

***Corresponding author:**
Dr. rer. nat. Susanne Gaul
Klinik und Poliklinik für Kardiologie, Universitätsklinikum Leipzig,
Johannisallee 30, 04103 Leipzig, Germany
Email: Susanne.Gaul@medizin.uni-leipzig.de
Fon: +49 341 9725844

https://orcid.org/0000-0003-4780-1844

Content:

Supplementary Methods
Supplementary Figures S1-S2 and Figure legend
Resources table
Original uncropped western blots

Supplemental Methods

Cell culture

THP1 monocytic cell line (DSMZ, Cat.No: ACC16) was cultured in RPMI 1640 medium supplemented with 10% (v/v) FCS, 100 U/ml penicillin and 100 mg/ml streptomycin. THP-1 monocytes were differentiated to macrophages for 4 days with phorbol myristate acetate PMA (100 nM). The THP1 ASC-GFP reporter monocytic cell line (Invivogen, Cat.No. thp-ascgfp) was cultured in RPMI 1640, 2 mM L-glutamine, 25 mM HEPES, 10% heat-inactivated fetal bovine serum, 100 µg/ml Normocin, Pen-Strep (100 U/ml-100 µg/ml). Zeocin was added as selective antibiotic (100 mg/ml).

Isolation of ASC-GFP inflammaome specks from THP ASC-GFP cells

THP1 ASC-GFP reporter cells (1-6x 10^7 cells) were stimulated with LPS (100ng/ml, 3h) und Nigericin (10µM, 30 min) before they were used for the isolation of ASC-GFP specks as described previously by Martín-Sánchez et al [19]. ASC-GFP specks were counted using the Keyence BZ-X810 microscope.

Confocal microscopy of internalized ASC- GFP specks

HCASMC were seeded at a density of 1x 10^4 cells per cm^2 on 25-mm coverslips 24 h before the start of the experiment. HCASMC were treated with extracellular ASC- GFP specks for 4 h (3:1 particles/ cell). Coverslips were then washed with HBS and incubated with CellMask Deep Red plasma membrane stain (Invitrogen, Carlsbad, California, USA) for 10 min at 37°C according to the manufacturer's protocol. Coverslips were mounted and imaged using a Leica DMI8/SP8 confocal laser scanning microscope (Leica Microsystems, Wetzlar, Germany). Pinhole adjustment was set to 1 Airy unit. For YFP excitation a 488 nm and for CellMask Deep Red plasma membrane stain a 638 nm Laser was used. Emission filters were adjusted accordingly. Images were analyzed using LasX (Leica Microsystems, Wetzlar, Germany) and ImageJ 1.53e Java 1.8.0. version (https://imagej.nih.gov/ij/) .

Immunoblot analysis

HCASMC were stimulated with ASC- GFP specks for 4h and homogenized in RIPA buffer (Cell Signaling, USA) containing protease inhibitor cocktail HALT (ThermoFisher, USA). For immunoblot analysis 20- 30 µg of protein lysate was resolved on Any kD Mini-PROTEAN TGX Precast polyacrylamide gels (Biorad, Hercules, CA, USA), transferred to nitrocellulose membrane, blocked in 5% Blotting-grade blocker (Biorad) and incubated with appropriate primary antibodies. Anti- IL1β (1:1000, Abcam), anti- caspase-1 p20 Bally-1 (Adipogen), anti-NLRP3 NBP1 (1:1000, NBP1-77080 Novus Biological), anti-Gasdermin D (L60) (1:1000, Cell Signaling) and anti- β- Actin (1:10,000, Abgent) were incubated over-night. β- Actin was used for normalization. Membranes were incubated with peroxidase-conjugated secondary antibody (DAKO, USA). Protein bands were visualized with the enhanced chemiluminescence (Pico or Femto, Pierce ThermoFisher Scientific, Waltham, MA USA) reagent and digitized using iBright FL1500 Imaging System (ThermoFisher, USA).

IL-1β ELISA

Supernatant of THP-1 macrophages were used for the analysis of released IL-1β using the Human IL-1 beta/IL-1F2 Quantikine ELISA Kit (R&D, DLB50).

References:

19. Martin-Sanchez, F., Gomez, A.I. & Pelegrin, P. Isolation of Particles of Recombinant ASC and NLRP3. *Bio-protocol* **5** (2015).

Supplementary Figures

Supplementary Figure S1

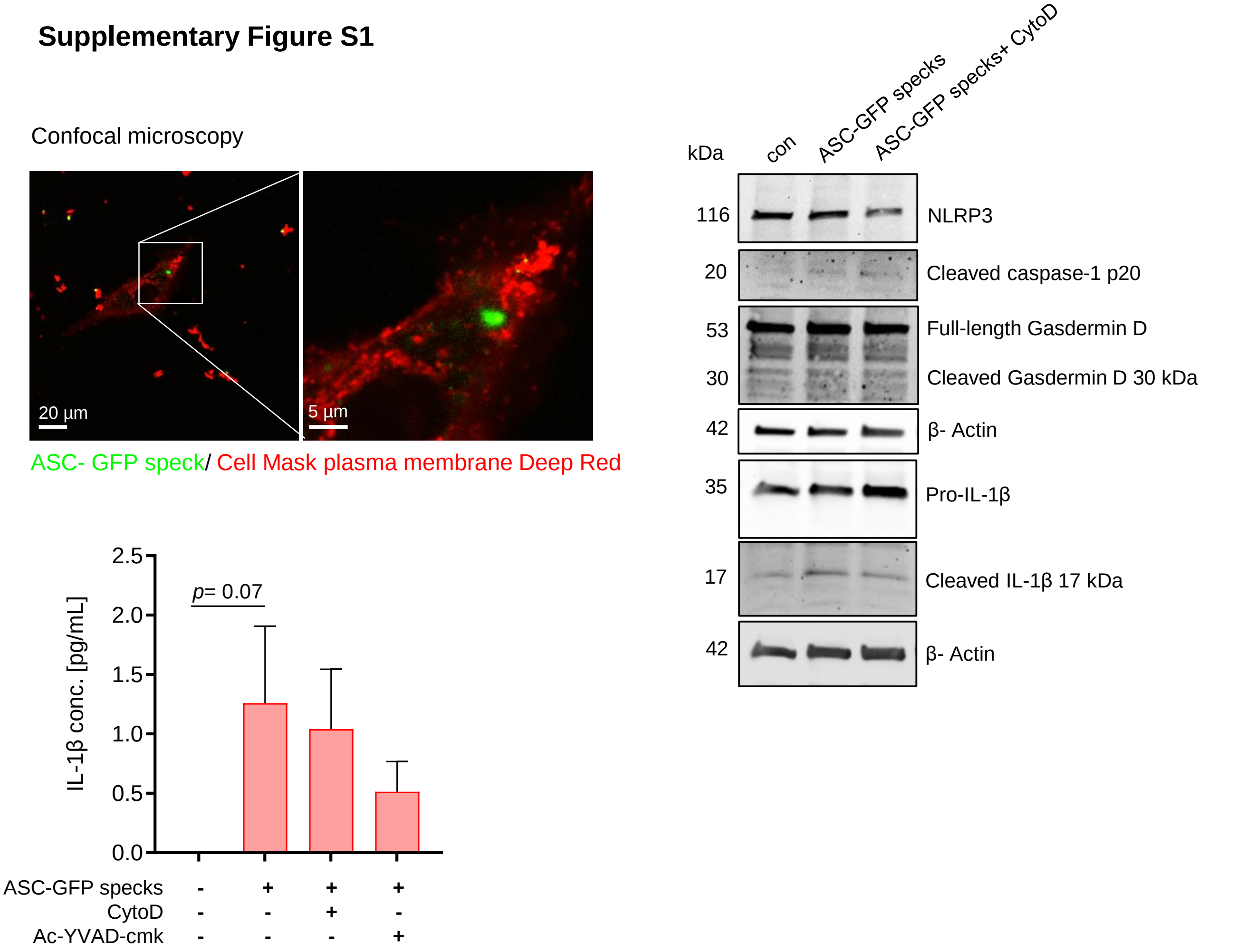

Supplementary Figure S1. Internalization of extracellular ASC- GFP specks (purified from inflammasome activated THP1 ASC-GFP reporter cells (green). HCASMC were incubated for 4 h. Uptake was confirmed via confocal microscopy. Plasma membrane staining (red) was carried out using CellMask Deep Red (scale bar: 20 µm). Micrographs depicting internalization and subcellular localisation of ASC-GFP specks are shown in the zoom-in image (scale bar: 5 µm) (left panel). Western blot of HCASMC lysate after treatment with extracellular ASC- GFP specks (3:1 specks/cell) for 4 h with our without pre-incubation of Cytochalasin D (CytoD, 4 µM) for 30 min. NLRP3, pro-IL-1β and mature cleaved IL-1β 17 kDa, activated caspase-1 p20 (4 h) and Gasdermin D (full-length and cleaved 30 kDa N-terminal fragment) are shown. Supernatant (10x concentrated) of HCASMC treated with ASC-GFP specks for 4 h with or without cytochalasin D (CytoD, 4 µM, 30 min) or caspase-1 inhibitor (25 µg/ml) was used for IL-1β ELISA (N= 3). Groups were compared using One-Way ANOVA and uncorrected Fisher´s LSD post hoc test.

Supplementary Figure S2

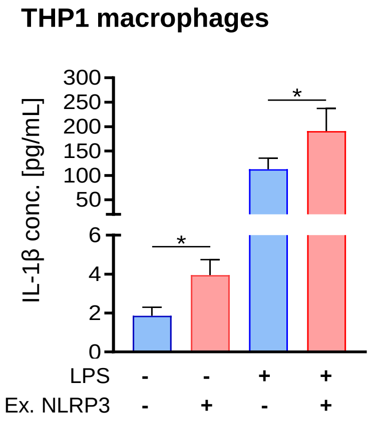

Supplementary Figure S2. IL- 1β (pg/mL) release from THP-1 macrophages primed with or without LPS (100 ng/ml, 3 h) and co- incubation with extracellular NLRP3- YFP inflammasomes for 24 h (N= 6). Data are represented as mean± SEM. Two groups were analyzed by unpaired, two-tailed Student´s T-test (*p< 0.05).

Resources Table

Antibodies

Target antigen	Vendor or Source	Catalog #	Working concentration
YFP (GFP)	Abcam	ab6556	2.5 ug/ml
NLRP3/NALP3	Novus Biologicals	NBP1-77080	1:1000
Rabbit IgG (H+L) Secondary Antibody, Alexa Fluor 647	ThermoFisher	A-21245	1:1000
IL-1β	Abcam	ab9722	1:1000
caspase-1 p20 Bally-1	Adipogen	AG-20B-0048-C100	1:500
ASC, pAb (AL177)	Adipogen	AG-25B-0006-C100	1:1000
β- Actin	Abgent	WA-AM1829B	1:10,000
Fibronectin	Santa Cruz	sc-8422	1 µg/ml
Gasdermin D (L60)	Cell Signaling	#93709	1:1000
Goat IgG anti-Mouse IgG (H+L)-Cy3	Dianova	115-165-146	1:1000

DNA/cDNA Clones

Clone Name	Sequence	Source
NLRP3 Taqman Probe Assay	Hs00918082_m1, Lot: 1843655	ThermoFisher Scientific
IL1b Taqman Probe Assay	Hs01555410_m1, Lot: P181218-003H08	ThermoFisher Scientific
B2M	FW: GAGGCTATCCAGCGTACTCCA<br>RV: GGCAGGCATACTCATCTTTT	Biomers
TBP	FW: CCACTCACAGACTCTCACAAC<br>RV: CTGCGGTACAATCCCAGAACT	Biomers
ICAM1	FW: TGATGGGCAGTCAACAGCTA<br>RV: GGGTAAGGTTCTTGCCCACT	Biomers
Spon1	FW: CCCAAGTCAGAGGGCTACTG<br>RV: GGTTCCCGGCTTGTAGAAGT	Biomers
GFPT2	FW: TTGGTCGAGAGAGTCATTCAGC<br>RV: AAGATAGGGATCTGTTCTGTGGA	Biomers
Nup210	FW: ATGCCTTCCGATCAGTACGAG<br>RV: CGACCACGTAGATAGTGCTGT	Biomers
RPLP0	FW: TCGACAATGGCAGCATCTAC<br>RV: ATCCGTCTCCACAGACAAGG	Sigma
CADM1	FW: ATGGCGAGTGTAGTGCTGC<br>RV: GATCACTGTCACGTCTTTCGT	Biomers

Cultured Cells

Name	Vendor or Source
Primary human coronary artery smooth muscle cells (HCASMC)	Lonza
THP1 monocytic cell line	DSMZ
stable mutant NLRP3 (p.D303N)-YFP HEK cell line	Provided by Pablo Pelegrin[1]
HEK 293 cells	DSMZ
THP1 ASC-GFP reporter cells	Invivogen

Datasets

Description	Source	URL
NCBI's Gene Expression Omnibus[2]	Ayari et al. [3]	https://www.ncbi.nlm.nih.gov/sites/GDSbrowser?acc=GDS5083#details DataSet Record GDS5083

Other reagents

Description	Source
Caspase-1 inhibitor Ac-YVAD-cmk, 25 µg/ml	Invivogen
NFκB inhibitor IKK-16 (2 µM)	Selleckchem
Cytochalasin D (4 µM), PHZ1063	Invitrogen
Pierce LDH Cytotoxicity Assay	Pierce, ThermoFisher Scientific
QCM Chemotaxis Cell Migration Assay, CLS3422-48EA	Merck
PDGF (10 ng/ml), 100-14B	PeproTech
Actinomycin D (5 µg/ml), A1410	Merck
Human IL-1 beta/IL-1F2 Quantikine ELISA Kit, DLB50	R&D
CellMask Deep Red Plasma membrane Stain, C10046	Invitrogen
Hoechst 33342	Immunochemistry

References

1. Baroja-Mazo A, Martin-Sanchez F, Gomez AI, et al. The NLRP3 inflammasome is released as a particulate danger signal that amplifies the inflammatory response. *Nat Immunol*. 2014;15(8):738-748. doi:10.1038/ni.2919.
2. Barrett T, Wilhite SE, Ledoux P, et al. NCBI GEO: archive for functional genomics data sets--update. *Nucleic Acids Res*. 2013;41(Database issue):D991-5. doi:10.1093/nar/gks1193.
3. Ayari H, Bricca G. Identification of two genes potentially associated in iron-heme homeostasis in human carotid plaque using microarray analysis. *J Biosci*. 2013;38(2):311-315. doi:10.1007/s12038-013-9310-2.

Original uncropped western blots

Figure 1F

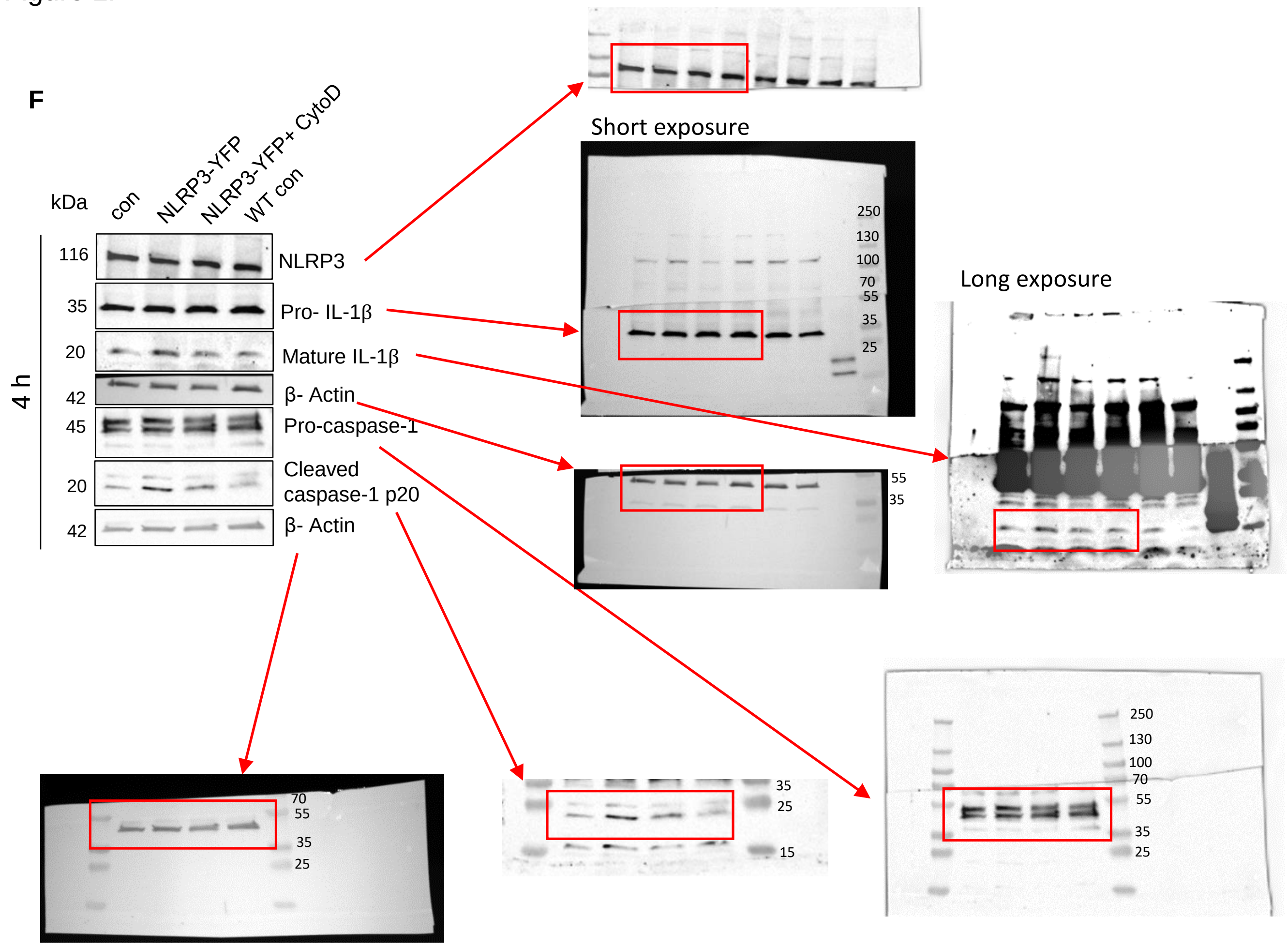

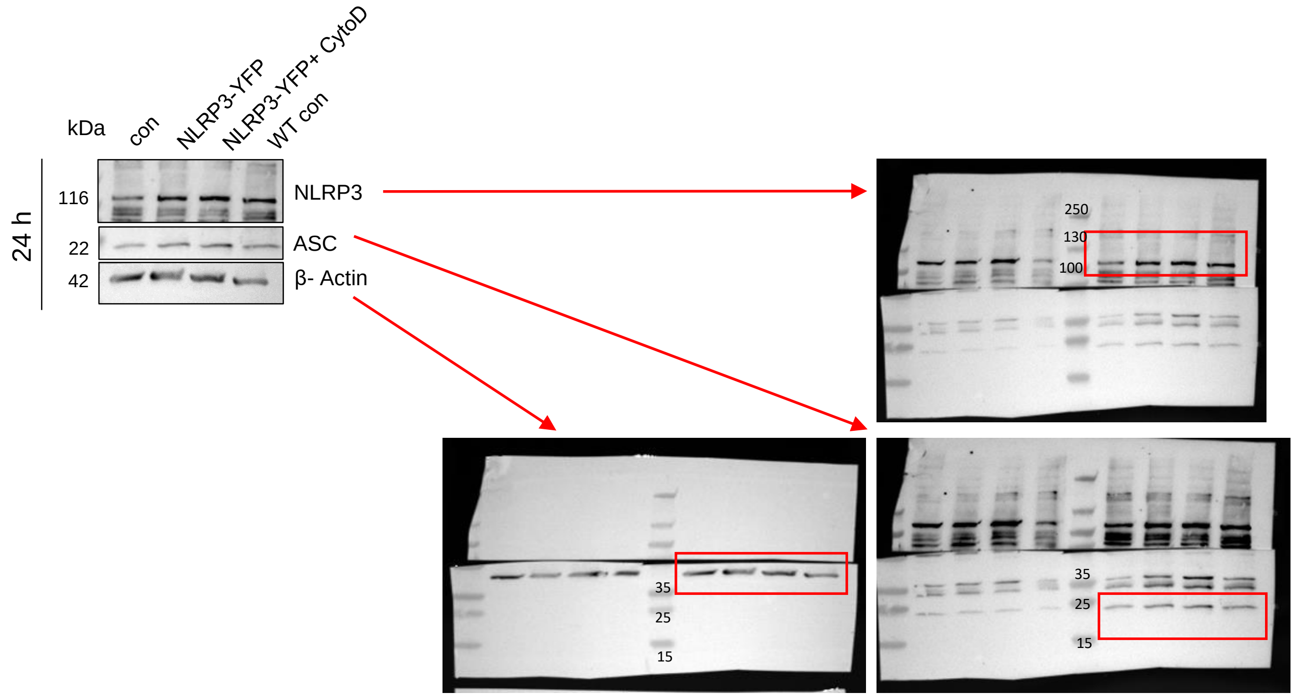

Original uncropped images

Figure 1M

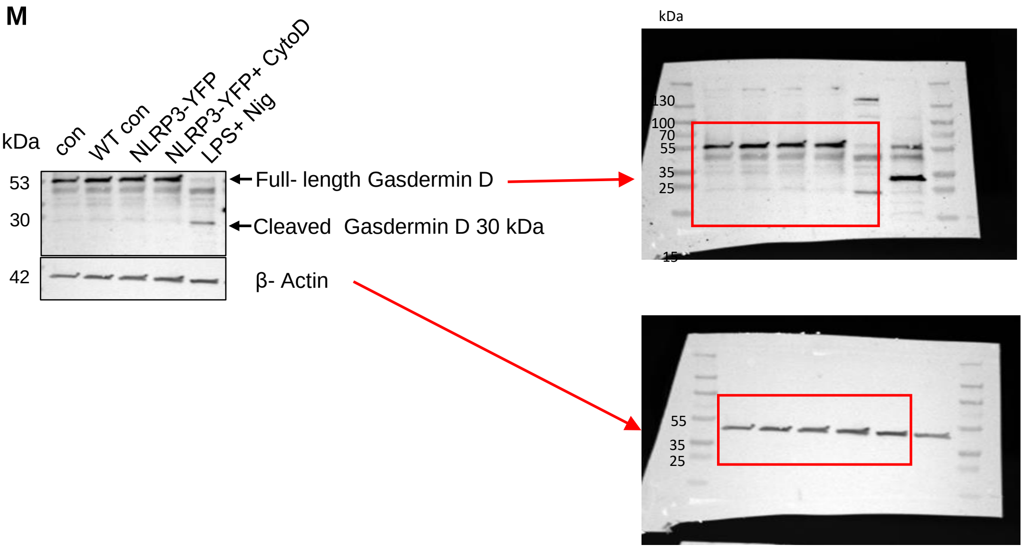

Original uncropped images

Supplementary Figure S1

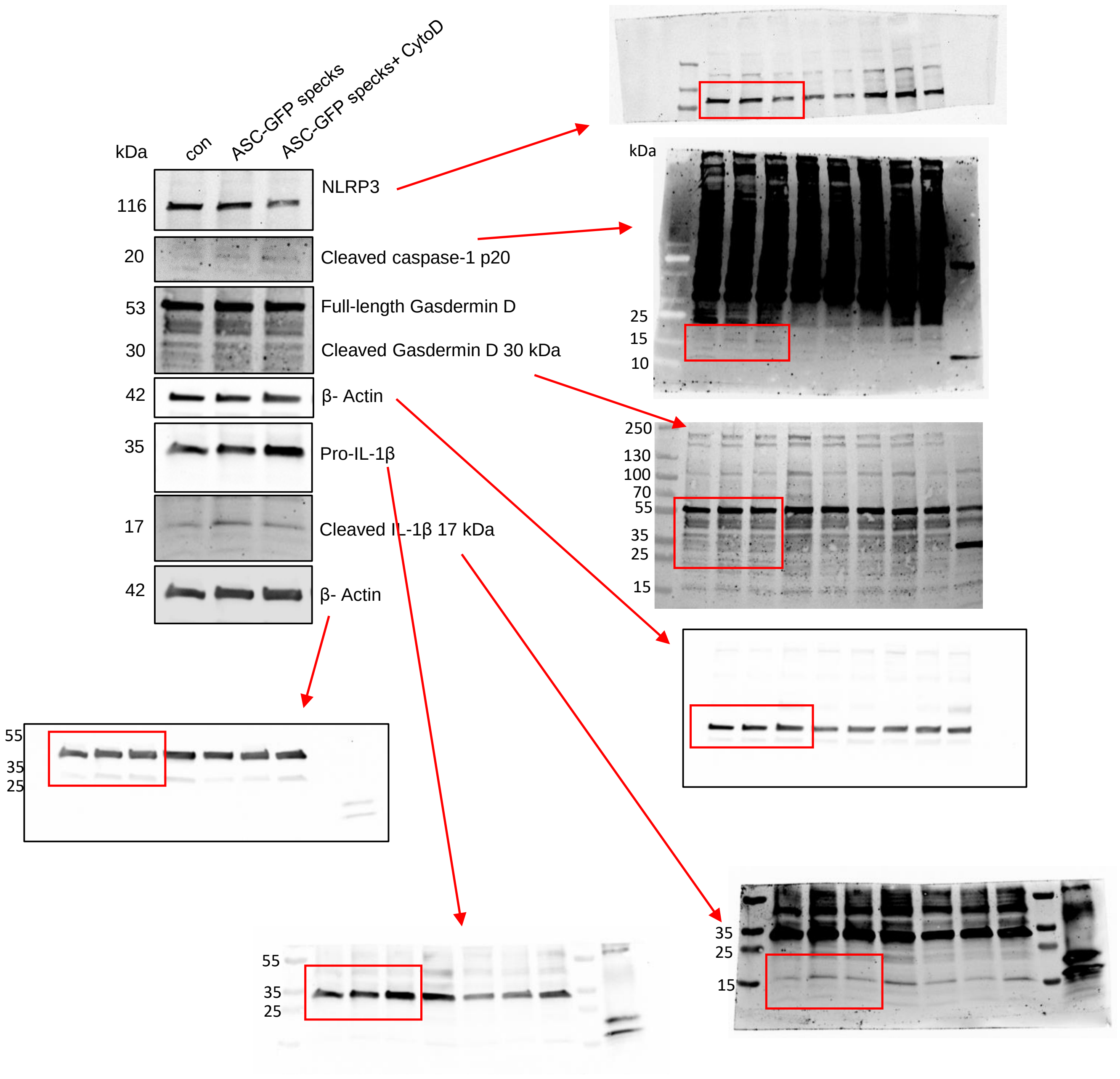

Supplement: Supplementary file 1 — Supplementary file. [file 41598_2021_94314_MOESM1_ESM.pdf]
